# A novel single-channel edge computing LoRa gateway for real-time confirmed messaging

**DOI:** 10.1038/s41598-024-59058-8

**Published:** 2024-04-10

**Authors:** Chen Zhong, Xianzhong Nie

**Affiliations:** 1 The Department of Economics and Information Management, Shanghai University of Finance and Economics Zhejiang College, Jinhua, 321013 China; 2 The Department of Research and Development, Zhejiang Huiju Intelligent IoT Co, Hangzhou, 311100 China

**Keywords:** Engineering, Mathematics and computing

## Abstract

LoRaWAN has become the technology of choice for increasing Internet of Things applications owing to its long range and low power consumption characteristics. However, in the uplink confirmed messaging cases, the entire retransmission could take several seconds, so it cannot be used in scenarios that require rapid confirmed messaging, such as emergency alerting and real-time controlling applications. Nevertheless, there has been limited work targeting this issue. This study presents a novel LoRaWAN gateway using edge computing to expedite the confirmed messaging process by generating the acknowledgment (ACK) locally, so that the confirmed messaging time can be significantly reduced. Additionally, the resource utilization of the network server can also be decreased due to the use of edge computing. We verified the effectiveness of our solution through extensive simulations and experiments. The confirmed messaging time between the end nodes and the gateway averaged 43 ms for a maximum of 2 retransmissions. With the adoption of edge computing on the gateway, the network server’s central processing unit (CPU), memory, and bandwidth peak utilization decrease from 53.51 to 39.46, 73.88 to 72.11%, and 4422.68 kbps to 3271.27 kbps, respectively. In addition, the network server’s system load decreases from 2.15 to 1.69, while the gateway cost is reduced by almost $\$$38 compared to the benchmark products.

## Introduction

Devices in the Internet of Things (IoT)^1^ can be connected via Wi-Fi^2^, Bluetooth^3^, and Zigbee^4^. With the rapid development of low power wide area networks (LPWAN) technology, LoRaWAN^5^ has become the first choice for many IoT applications such as smart cities, smart homes, and industrial control. LoRaWAN has three distinct operating classes (A, B, and C) to support various applications. The class A mode of LoRaWAN is widely used in various battery-powered end nodes due to its low power consumption. In this mode, the end node opens the receive window RX1 and RX2 for a fixed time slice after sending a frame of data, in which it can receive a message or an ACK from the cloud-based network server (NS). Since the receive window is opened for a certain period of time, this model is not suitable for real-time demanding scenarios. For this reason, the class C mode is used in high real-time scenarios, where the receive window is always open, making it impossible for the nodes to go to sleep, and thus the power consumption is high. The class B mode allocates a receiving window for each node, and thus the downlink information can reach the node faster than class A. However, the energy consumption of class B is higher than class A because the node opens the receiving window periodically.

Despite the success of LoRaWAN in many domains, some issues exist in some scenarios, particularly where local deployments and rapid acknowledgment are required. The LoRaWAN specification defines two receive windows (RX1 and RX2) for the end nodes to receive downlink data and ACK. In the case of high network load, confirmed messages need to be retransmitted multiple times due to considerable packet collisions. Since the ACK is generated by the cloud-based NS, and if multiple retransmissions are performed, the confirmed messaging time will be increased by more than 2 seconds for each retransmission attempt. In some applications involving urgent messages or real-time response, such as a fire alert, or a light control command, these uplink messages should reach the cloud-based network server within 1 s. However, the LoRaWAN standard and the state-of-the-art solutions can not fulfill such real-time requirements. In addition, the CPU, memory, and bandwidth^6^ usage of the network server is very high when numerous nodes transmit confirmed packets simultaneously, which demands more computing and communication resources for the cloud-based network server.

The contributions of this study are as follows:

- We proposed a novel single-channel gateway design by selecting an appropriate microcontroller unit (MCU) and LoRa module to significantly reduce the cost. An MCU is a compact integrated circuit designed to govern a specific operation in an embedded system. A typical microcontroller includes a processor, memory, and input/output peripherals on a single chip. The cost of MCUs can vary widely, and the details of the gateway cost calculation are illustrated in Table 1.
- We designed an edge-computing LoRaWAN gateway to attain real-time confirmed messaging. This edge computing capabilities allow for real-time data transmission, processing, and performing security mechanisms directly at the network’s edge, reducing the need for cloud server resources in terms of CPU, memory, bandwidth, and system load.
- We experimentally validated the confirmed messaging time reduction to the millisecond level for edge-computing LoRaWAN gateways and reduced the network server resource usages^7^.

**Table 1:** The cost of main components of traditional and proposed LoRaWAN gateways.

	Raspberry Pi	MTK7688	SX1278	SX1302	SX1255	Total cost
	$14	$8	$1.5	$18	$8	
Traditional LoRaWAN gateway	$$\checkmark $$			$$\checkmark $$	$$\checkmark $$	$48
Proposed edge-computing LoRaWAN gateway		$$\checkmark $$	$$\checkmark $$			$9.5

To improve readability, Table 2 summarizes the abbreviations used in this article.

**Table 2:** List of abbreviations.

ACK	Acknowledgment	CPU	Central Process Unit
IoT	Internet of Things	LPWAN	Low Power Wide Area Networks
NS	Network Server	MCU	Microcontroller Unit
MQTT	Message queuing telemetry transport	DC	Direct Current
SPI	Serial Peripheral Interface	RF	Radio Frequency
PHY	Physical Layer	PCB	Printed Circuit Board
PRR	Packet Reception Ratio	RSSI	Received Signal Strength Indicator
SNR	Signal-to-noise Ratio	QR	Quick-response

## Related work

Traditional LoRaWAN employs gateways for transparent transmission, and all data process is conducted on the cloud-based network server, which results in increased communication latency, network congestion, and network server load. In^8^, a novel distributed computing model is introduced and a latency-aware algorithm is used for edge node task processing. Computing functions such as data preprocessing and machine learning model training are incorporated into the LoRa gateway, which effectively improves network reliability. Compared to the conventional LoRaWAN, it reduces the CPU and bandwidth usage without affecting the system throughput. However, the experiments were conducted under network simulations, rather than using real-world LoRa end nodes. Our proposed method advances beyond simulations by implementing a novel single-channel gateway, which is experimentally validated to reduce confirmed messaging time to the millisecond level and decrease network server resource usage, thereby offering a practical solution for real-time applications.

In^9^, a low-cost LoRa and edge-computing-based system architecture was applied to assess forest fire occurrence. Nonetheless, this study provides limited insights into the specific implementation of the edge gateway, primarily focusing on the description and benefits of the application system, and lacks a detailed explanation of the advantages of the edge computing gateway. Furthermore, hardware components such as the MCU and LoRa modules used in the LoRa gateway are not exhaustively detailed, making it challenging to evaluate their cost. While our method provides a concrete design and experimental validation of an edge-computing LoRaWAN gateway, emphasizing real-time messaging and cost efficiency, thus offering a more comprehensive solution with potential applicability in a wider range of scenarios including emergency response.

The authors of^10^ presented an adaptive spreading factor selection scheme with an iterative spreading factor detection algorithm to reduce erroneous spreading factor selection for single-channel LoRa networks. Nevertheless, the authors briefly mentioned the results of other studies without providing an in-depth analysis. Regarding the experimental setup, the authors mentioned the use of a LoRa modem connected to a Raspberry Pi but provided limited specifications about other hardware components and the experimental environment. Additionally, although this study evaluates the performance of single-hop and multi-hop LoRa networks, it lacks a comprehensive analysis of the impact of different network topologies and sizes. As a comparison, our proposed method is based on low-cost hardware components which is an advantage for large-scale applications of single-channel LoRa networks.

The authors of^11^ suggested to use of multiple single-channel gateways to achieve fair transmission for all end nodes in the network by dynamically adjusting the channel allocation. However, this paper did not explicitly describe the MCU used, and it did not implement edge computing into the gateways. This drawback may lead to longer confirmed messaging time, rendering them unsuitable for emergency applications or scenarios requiring high real-time responses. Our proposed gateway design incorporates edge computing to directly acknowledge messages, significantly reducing confirmed messaging time and demonstrating a clear advantage in scenarios requiring rapid responses.

In^12^, LoRaWAN and Wi-Fi protocols were heterogeneously integrated into farm environment monitoring and edge computing technology was introduced to improve the transmission distance and farmland monitoring range. However, the gateway adopts Raspberry Pi as the hardware platform of the IoT gateway as well and the LoRa chip adopts SX1302 and two SX1255, which makes the manufacturing cost of the gateway high. Furthermore, based on the standard LoRaWAN protocol, the confirmed messaging time could still reaches several seconds although edge computing is adopted. Our proposed method emphasizes cost-efficiency and real-time messaging, showcasing a practical implementation that could potentially offer better scalability and application-specific adaptability at a lower cost.

A proof-of-concept of an edge-assisted IoT-based architecture was designed and implemented to optimize existing LoRa-based IoT applications through edge computing to achieve higher data rates, better scalability, and lower latency in^13^. Similarly, in^14^, an effective monitoring system model for agricultural facilities was created using edge computing and artificial intelligence methods. The machine learning-based monitoring system helps farmers monitor plant conditions by predicting deviations from normal factors, irrigating plants efficiently, and optimizing the use of expensive chemicals. This edge computing and machine learning-based monitoring system can be applied to home greenhouse and industrial greenhouse networks. In^15^, energy management system data is compressed by edge computing techniques and communicated by LoRa networks to a power operator at a given time with minimal energy consumption. These works illustrate the potential of application layer edge computing in optimizing LoRa-based IoT applications. In^16^, a long-range, energy-efficient vision node called EdgeEye is introduced for long-term edge computing. EdgeEye utilizes a low-power processor, GAP8, and a low-power camera to enable a smart IoT device that can continue to work for many years and be powered by a battery. The system architecture of EdgeEye, the design and performance evaluation of convolutional neural networks, the estimation of energy consumption and battery life, and the application of edge computing in IoT devices are also described in detail. A landslide monitoring and early warning system based on edge computing is introduced in^17^, including the common transmission methods for communication of geohazard monitoring devices, the analysis and comparison of the characteristics and advantages of multiple communication technologies, and the design and development of the intelligent superposition-triggered monitoring model based on the multi-parameter joint-triggered intelligent algorithm. These studies implement edge computing at the application layer, aiming to enhance specific IoT applications through localized data processing and decision-making. While beneficial for targeted use cases, this approach inherently limits the scope of improvement to the application layer, which may not universally optimize network performance across all potential IoT use cases. Our proposed method applies edge computing at the data link layer, which is a more foundational level within the network stack. This not only ensures that the enhancements are applicable across a wider range of IoT scenarios but also addresses systemic inefficiencies that are not application-specific. The universal applicability of our approach makes it a fundamentally more robust solution for enhancing IoT networks through edge computing.

## The design of system and the gateway

### System architecture

**Figure 1:**
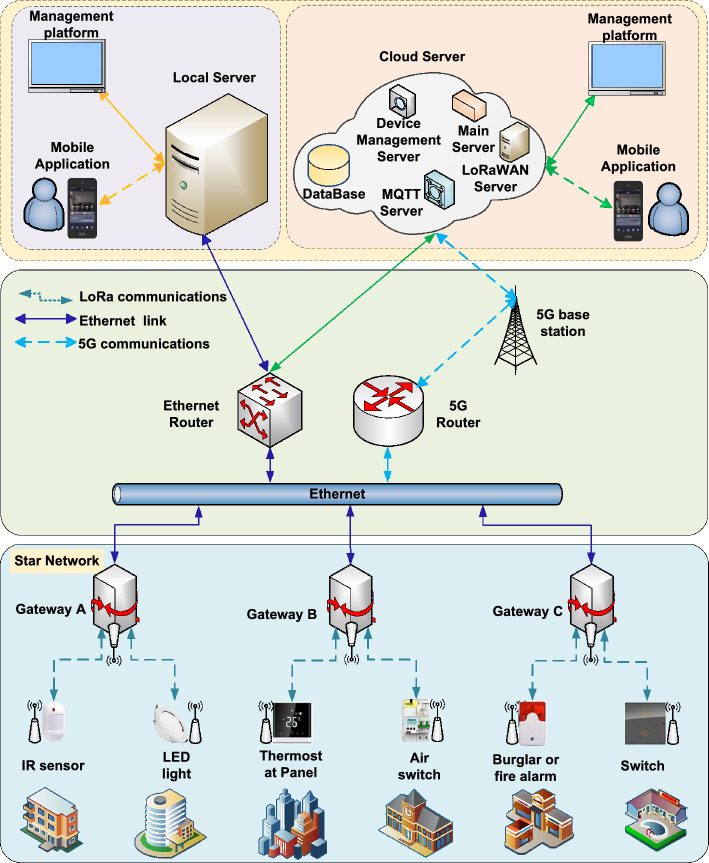
System architecture which comprises: a mobile application, a management platform, a server (cloud-based or local), LoRa gateways, and end nodes. Both the mobile application and management platform enable users to operate the end nodes, monitor their status, and receive messages.

Figure 1 illustrates the system architecture, which comprises various components: a mobile application, management platform, server (either cloud-based server or local server), LoRa gateway, and end nodes. Both the mobile application and management platform enable users to operate the end nodes, monitor their status, and receive uplink messages. The server is a pivotal component of this system, which includes a main server, a LoRaWAN server, a message queuing telemetry transport (MQTT)^18^ server, a device management server, and a database. It offers functions such as data storage, processing, network services, and security. The server can be deployed either locally or cloud-based. The MQTT protocol is used to connect the server and the gateway.

The LoRa gateway plays a critical role in the architecture, serving as a central hub for data relay, processing, and security. The gateway and server are connected via Ethernet or 5G. A star network^19^ topology with advantages such as easy maintainability, high reliability, and scalability is used between the gateways and the end nodes.

End nodes report data to and execute the control commands from the server. These end nodes include, for instance, smart switches, motion infrared sensors, microwave radar sensors, and magnetic sensors. The versatility of the end nodes makes it suitable for a wide range of applications, including smart homes^20^, building automation^21^, smart agriculture^22^, smart cities^23^, and environmental monitoring^24^.

### Electronics design

#### Overall design

**Figure 2:**
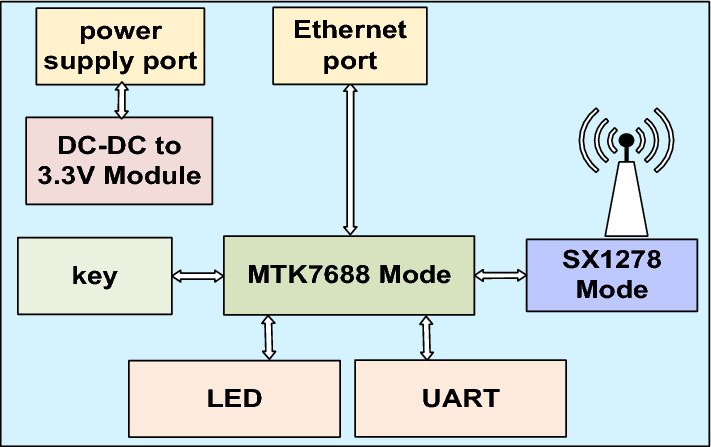
The overall hardware design of the gateway which comprises a DC-to-DC converter, MTK7688 module, SX1278 module, LEDs, universal asynchronous receiver/transmitter (UART), and keys.

We considered various factors during the gateway design, including user-friendliness, ease of installation, debugging, and maintenance, low cost, and low confirmed messaging time. Figure 2 illustrates the hardware design of the gateway. We employed a 12–24 V direct current to direct current (DC-to-DC) converter to provide the power for the gateway. The core control unit is the MTK7688^25^ module, which enables MQTT communication with the server via an Ethernet connection. The serial peripheral interface (SPI)^26^ is used to communicate with the SX1278^27^ module, which provides LoRa connections. Furthermore, the gateway sends debugging information through both the serial and Ethernet ports and illustrates 3 statuses via light-emitting diodes (LEDs), which are power supply, online or offline, and packet transmission and reception. The key is designed for resetting the gateway.

#### MTK7688 module

**Figure 3:**
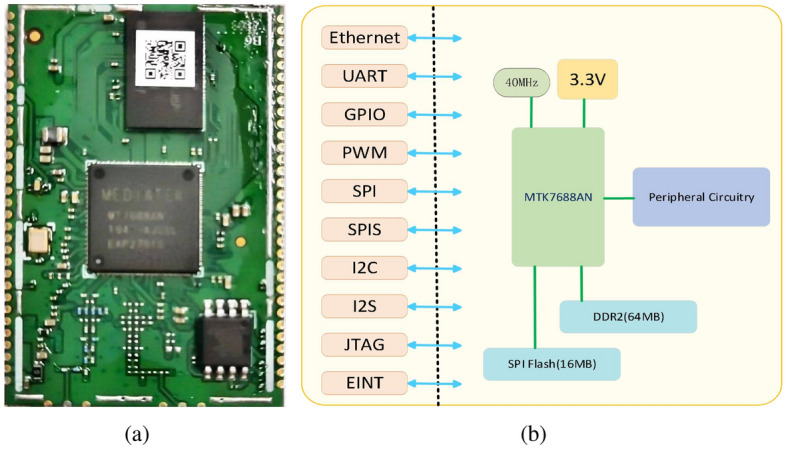
(**a**) Assembled MTK7688 module. (**b**) MTK7688 module which comprises a 40 MHz external crystal oscillator, flash and DDR2 memories, and a group of peripheral interfaces.

For the selection of a core control unit, the primary considerations are high clock frequency, adequate memory (i.e., for edge computing), and ease of use. We have selected the MTK7688 module from LILDA Group^28^, which is cost-effective at approximately $\$$8. Compared to alternatives like the Raspberry Pi^29^, which retails for $\$$14, the cost is reduced by $\$$6. Figure 3a shows the module. The core processing unit of the MTK7688 module utilizes an MT7688AN controller which integrates a 580 MHz MIPS®24KEc^TM^ CPU. Its clock signals are produced by an external 40 MHz crystal oscillator which is shown in Fig. 3b. The Nanya^30^ NT5TU32M16FG-AC^31^ chip provides 64 MB of double data rate 2 (DDR2) flash memory. For nonvolatile memory, we employed a 16 MB flash memory namely MXIC25L112835FM21-10G^32^ from Huabang^33^.

#### SX1278 module

**Figure 4:**
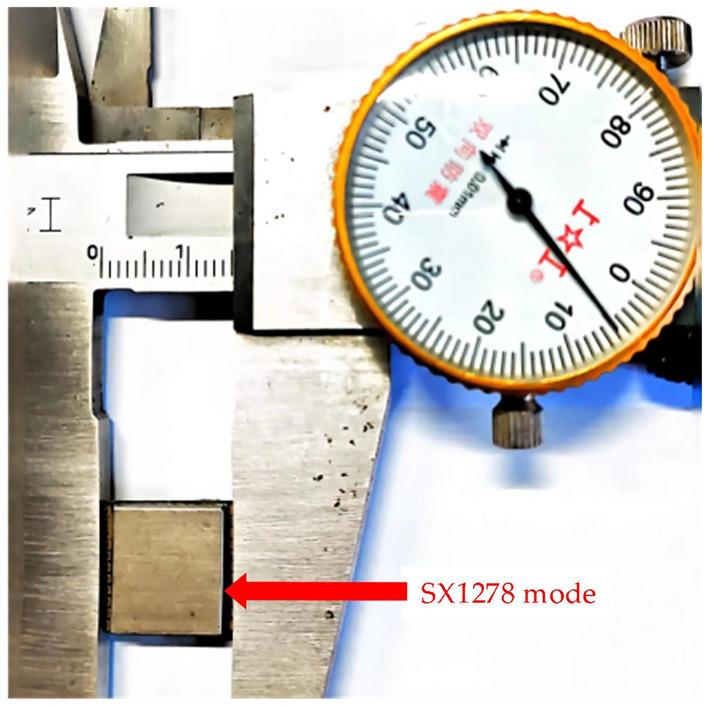
SX1278 module.

The SX1278 module from Nanjing Renyu^34^ enables a single-channel LoRa communication between the gateway and the end nodes, which is illustrated in Fig. 4. Its radio frequency (RF) circuitry is designed to match a 50 $\Omega$ impedance. Table 1 presents the relevant component pricing from Digikey^35^, revealing that SX1278 is available at a cost of only $\$$1.5, whereas the conventional multi-channel gateway comprises a digital baseband chip (i.e., SX1301 or SX1302^36^) with a cost of approximately $\$$18. Additionally, the cost of the two pieces of Multi-PHY mode transceiver (i.e. SX1255) is approximately $\$$16. Therefore, the cost of a SX1278-based single-channel gateway is less than one-twentieth of the multi-channel one. Since our novel gateway targets rapid confirmed messaging, in which an ACK is required for each uplink transmission, using the multi-channel gateway will not benefit the network. In addition, we chose to set the spreading factor, and bandwidth of the network to 7 and 500 kHz to attain the highest data rate, so that the confirmed messaging which involves multiple retransmissions can be completed as soon as possible (i.e. the lowest confirmed messaging time). However, boosting the data rate results shortened communication distance. Accordingly, the number of gateways deployed needs to be increased to retain the LoRa signal coverage compared to the conventional long-range multi-channel gateway. As a result, the cost of rapid confirmed messaging gateway should be substantially reduced to be affordable for practical use.

#### Ethernet

The Ethernet is used as the communication method between the gateway and the server, which is based on Pulse Electronics’s^37^ H1102NL physical layer (PHY)^38^ chip complying with the IEEE 802.3 specification^39^. This PHY chip employs differential signaling^40^ for the transmissions.

#### LED

**Figure 5:**
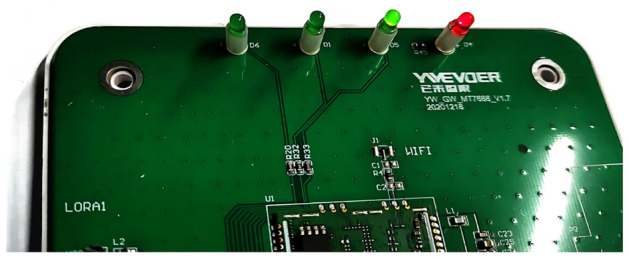
The arrangement of the Led on the PCB.

In the context of gateway applications, it is essential to employ LED indicators to provide a clear indication of the gateway’s current status which includes the power-on status, connectivity with the cloud-based server (i.e., online or offline), and the transmission and reception of the LoRa packets. Consequently, we have incorporated four LED indicators for these purposes. The arrangement of the LEDs on the PCB is shown in Fig. 5.

The first red LED signifies the power-on state, indicated by a constant illumination of the LED. The second green LED indicates the connectivity status, with the LED blinking and remaining unlit to denote the online, and offline status, respectively. The third green LED is designated for indicating the LoRa transmission and reception status, with one and two blinks, respectively. Lastly, the fourth green LED has been reserved for potential future use.

#### Printed circuit board design

**Figure 6:**
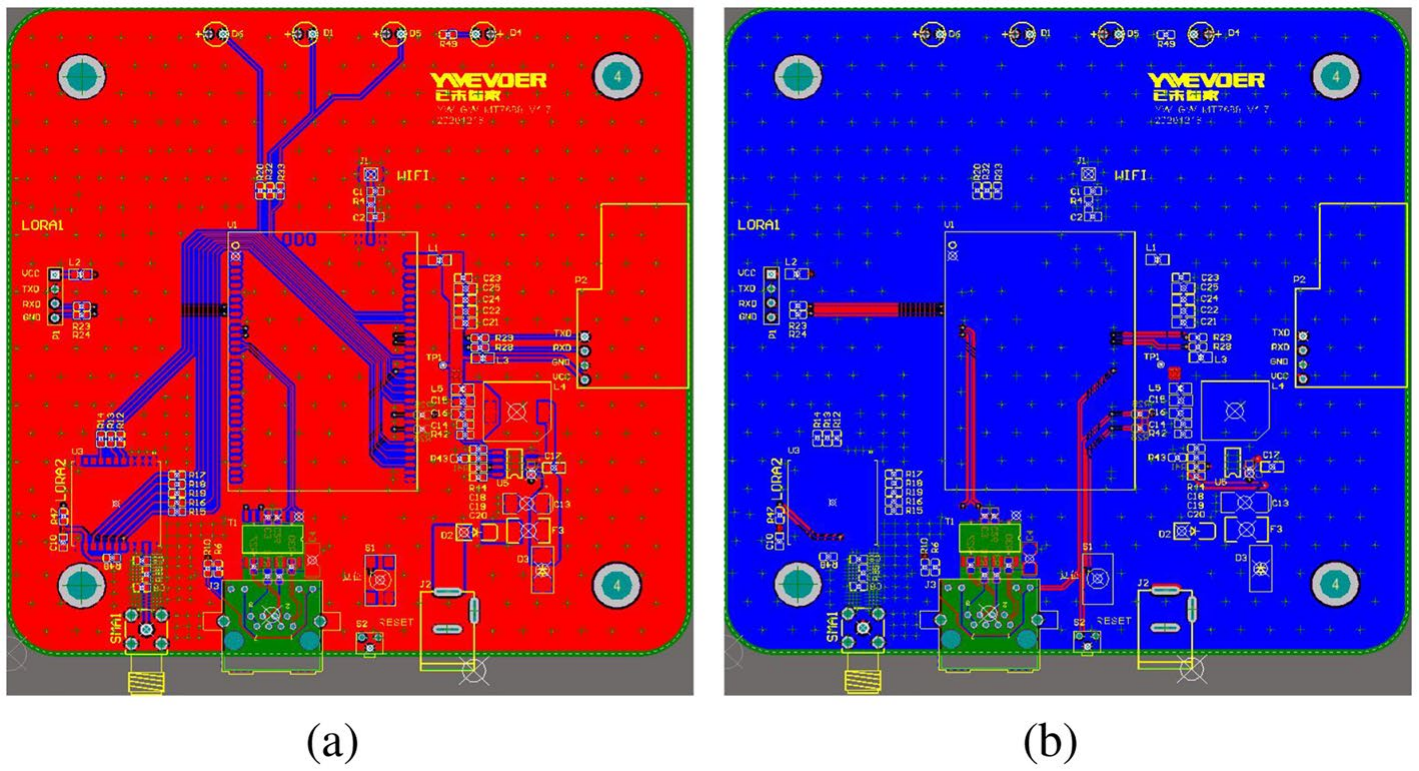
The PCB of Gateway. (**a**) Front side of the PCB design. (**b**) Back side of the PCB design.

Figure 6a,b depict the front and back sides of the circuit layout, respectively. To maintain impedance matching in the RF section and support the differential circuitry for the Ethernet, a four-layer printed circuit board (PCB) design was employed. The front and back layers contain the pads and lines that connect the pins between components, while the middle two layers are the power and ground layers.

### Functional design

#### Overall software design

**Figure 7:**
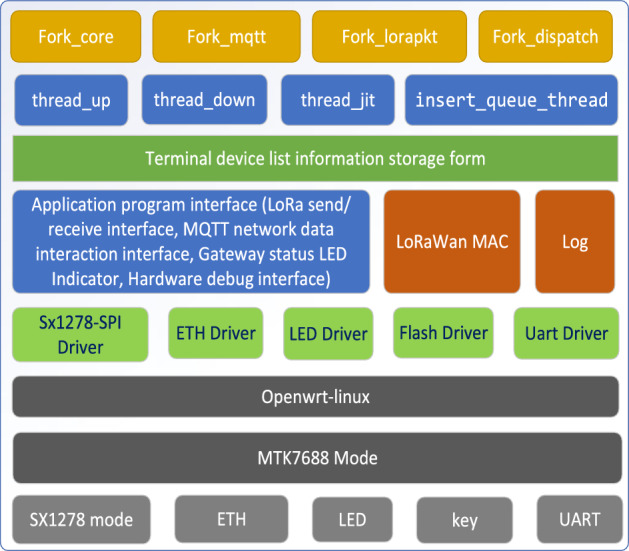
Software design block diagram, which comprises various components: Openwrt-Linux, hardware driver, four processes, four main threads, LoRaWAN MAC, MQTT client, Log, etc.

The block diagram of the software design is shown in Fig. 7. We chose the Openwrt-Linux^41^ as the operating system for our gateway, and implemented the hardware driver layer for SX1278, Ethernet, LEDs, and UARTs, and middleware components, such as LoRaWAN MAC and application program interface as shown in the figure. Task scheduling for the entire system was accomplished through four processes:

- Fork_core processes the application request data and generates responses to the end nodes according to user’s configurations.
- Fork_mqtt is used to connect to the server via MQTT.
- Fork_lorapkt manages LoRaWAN communication functions with the end nodes.
- Fork_dispatch acts as a message router between the MQTT and LoRaWAN, facilitating message transfers.

At the same time, we employed four main threads:

- Thread$\_$up processes packets received from the end nodes.
- Thread$\_$down handles the downlink data sent from the cloud-based server.
- Thread$\_$jit polls for pending downlink packets originated from the cloud-based server and dispatches them to the end nodes.
- insert$\_$queue$\_$thread is responsible for managing the incoming server’s data, processing it, and queuing it for distribution to end devices.

The MQTT protocol is used to connect the gateways and the network server due to its advantages of minimal packet overhead, efficient distribution to multiple clients, and reliable message queuing. However, the adoption of MQTT can not expedite the end-to-end delay for the LoRa confirmed messaging since the acknowledgment is still generated in the cloud-based network server, which results a long round trip time (RTT) for each transmission attempt.

#### Physical layer settings

The settings for the physical layers of the LoRa transceiver are listed in Table 3. Note that we chose the lowest LoRa spreading factor (i.e., 7) and the widest bandwidth (i.e., 500 kHz) to attain the highest data rate. The uplink receiving frequency of the gateway was set to a fixed frequency. On the contrary, the downlink frequency of the gateway was automatically adjusted according to the device address of the end nodes, which differs from the uplink frequency of the gateway. This arrangement prevents the problem of frequency conflict between the simultaneous downlink and uplink when the same one is used.

**Table 3:** LoRa physical layer settings.

Spreading factor	Bandwidth (kKHz)	Coding rate	Preambles	Frequency (MHz)	Transmit power (dBm)
7	500	4/5	8	470–510	17

#### Edge computing: acknowledging design

**Figure 8:**
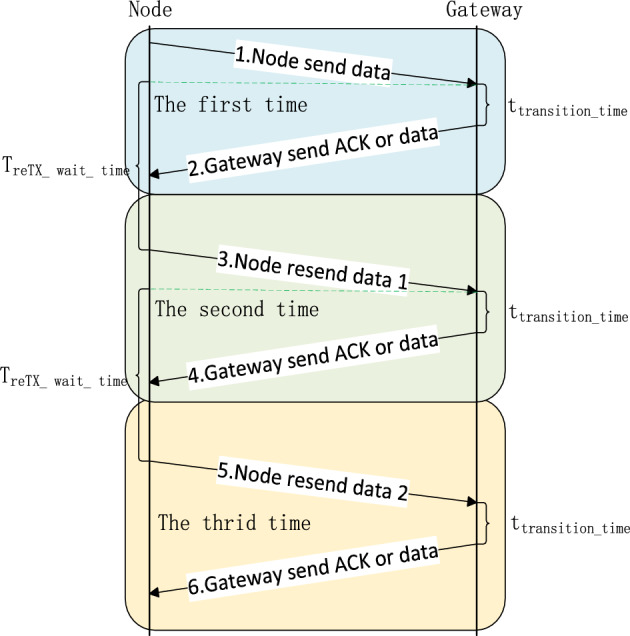
Gateway ACK timing design. The end node initiates its transmission and awaits the gateway’s ACK for a predefined time interval.

In LoRa communications, packet losses between end nodes and gateways sometimes occur. Therefore, a well-planned interaction-timing design can significantly enhance the reliability of LoRa communication. Figure 8 illustrates the timing when the gateway acknowledges the end node. The node initiates the transmission and starts a timer (i.e., $T_\mathrm {reTX\_wait\_time}$) after the end of its transmission.

Upon receiving data, the gateway performs the essential data verification checks and generates an ACK within a predefined $t_\mathrm {transition\_{time}}$. Subsequently, the ACK is transmitted to the node. If the node successfully receives the ACK before the $T_\mathrm {reTX\_wait\_time}$ timer expires, the transmission is completed. If not, the end node will start the first retransmission after the expiration of timer $T_\mathrm {reTX\_wait\_time}$. This process is repeated for up to three attempts. Then, the confirmed messaging is completed whether or not the node receives the ACK.

This approach differs from that of conventional LoRaWAN systems, in which the ACK response function is designed within the cloud-based server. In our design, the ACK response of the node occurs at the gateway. This modification reduces LoRa’s confirmed messaging time. In addition, it reduces the computing load and network bandwidth usage on the servers and thus, improves the scalability of the servers. In addition, the standard LoRaWAN ACK packet format is used in our design, which does not carry any payload if the net server do not have data to send to the end node.

#### Edge computing: gateway registration and replacement method

**Figure 9:**
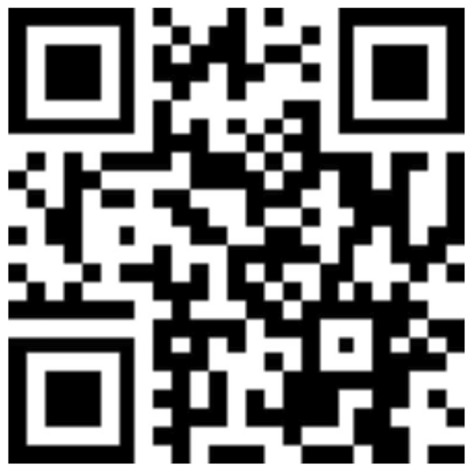
The QR code for the gateway ID 9F1000000001.

**Figure 10:**
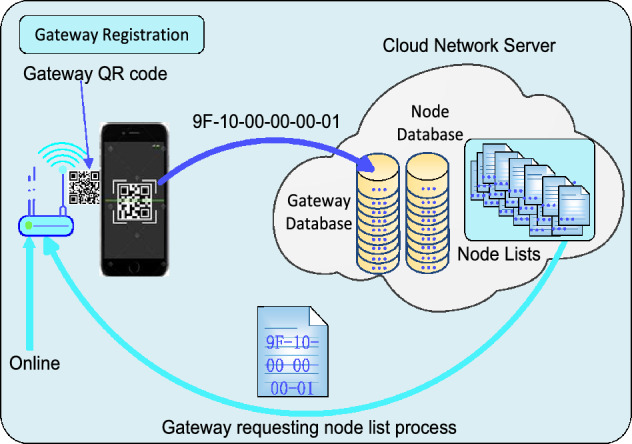
The gateway registration to the cloud-based network server through a QR code. Add the node list download process.

The use of quick-response (QR) code^42^ offers a quick, convenient, and secure method for integrating devices into IoT, which however, is not adopted by the conventional LoRaWAN. This approach minimized the risk of human error and expedited deployment processes. We designed 6 bytes to indicate the gateway’s identification (ID). An example of the QR code for the gateway ID 9F1000000001 is shown in Fig. 9. The process of gateway registration to the cloud-based network server is illustrated in Fig. 10. Its ID number is inserted into the gateway database in the server as a new device. After that, an associated node list is created and sent to the gateway when the node list is not vacant.

**Figure 11:**
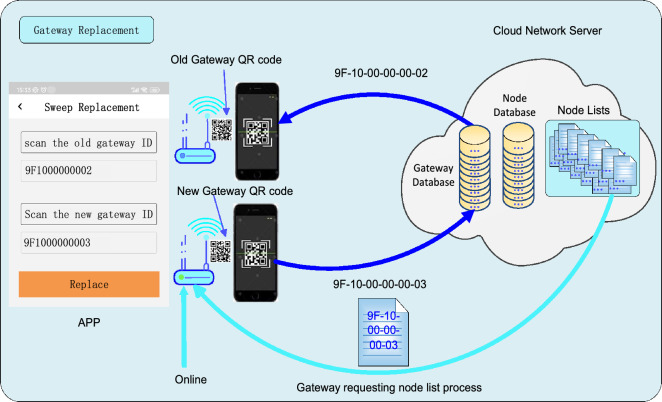
The gateway replacement function. To replace an old gateway, first scan its QR code, then scan the new gateway’s, and finally click the ‘Replace’ button in a dedicated mobile application.

It is conceivable that gateways inevitably experience malfunction or damage during their operational lifespan for various reasons. The simple way to handle this problem is to replace them with new ones. However, replacing the gateway with edge computing is more complicated than a transparent one (i.e., the conventional LoRaWAN gateway). To address this issue, we designed the gateway replacement function which is shown in Fig. 11. To replace an old gateway, first scan its QR code, then scan the new gateway’s, and finally click the ‘Replace’ button on the dedicated mobile application. With these user operations, the old gateway is substituted by the new one in the database of the network server. In addition, to ensure the seamless continuity of all original edge computing functions, the server transfers the node list associated with the old gateway to the new one once it is powered on and online.

#### Edge computing: node list synchronization

**Figure 12:**
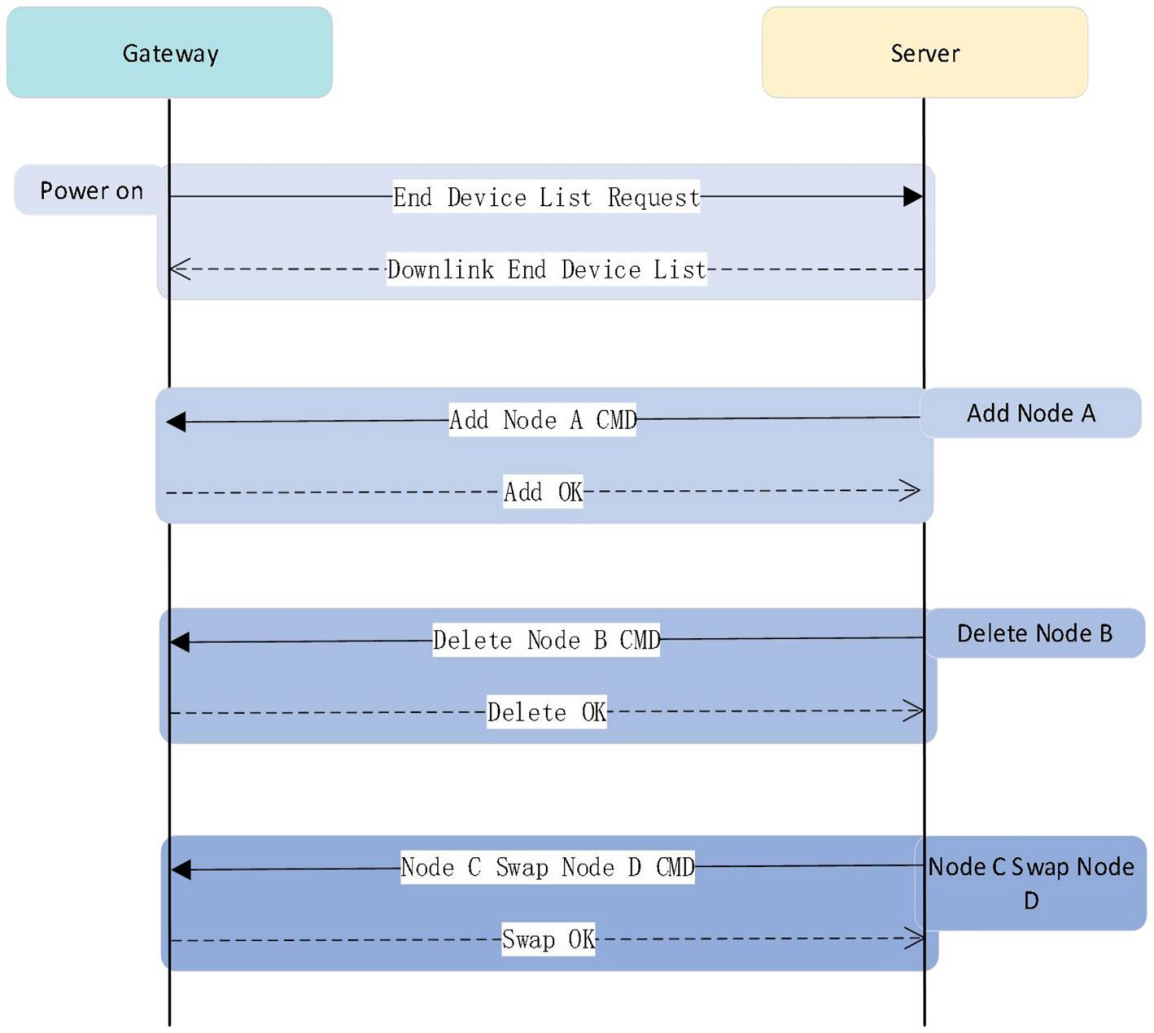
Process of synchronizing the node list on the gateway with the one on the network server.

In conventional LoRaWAN, the application server and the network server keep node information (i.e., a node list) while the gateway does not. Thus, the ACK is generated by the NS and disseminated by the gateway which is chosen by the NS. This will cause the end node to wait for the ACK for a long time after sending confirmed data, and the real-time confirmed messaging is not achievable. Thus, we suggest establishing an association between each end node and a chosen gateway by sending the node information to the gateway so that the ACK can be generated by the gateway directly. Such association is created and managed by the user and the list of associated nodes for each gateway is generated and sent by the network server if a gateway requests the list or the list is modified (i.e., the synchronization of node list between gateways and network server). The synchronization procedure that is shown in Fig. 12 is as follows:

- Upon connection to the server, the gateway initiates a request to the server for its node list. Subsequently, the server sends the relevant list to the gateway.
- In cases where a node (e.g., Node A) is added by the user, the server notifies the gateway, which then adds Node A to the node list.
- If the user removes a node (e.g., Node B) in the network server, the server notifies the gateway to perform the same operation. Then, the gateway conducts the removing and reports the result to the server.
- When a replacement operation is initiated by the user (e.g., use Node C to replace Node D), the server issues a replacement command to the gateway. After performing the replacement, the gateway will also report the result to the server.

This list synchronization process ensures that each gateway’s end node list is identical to the one on the network server-side.

#### Edge computing: security mechanisms

Traditional LoRaWAN devices use a unique device identifier (DevEUI) and a pre-shared key (i.e., AppKey) to authenticate themselves to the network. During the initial join process, the device and the network server perform a mutual authentication process, and the session keys (i.e., NwkSKey and AppSKey) are derived^43^. The NwkSKey is used for securing messages on the network layer, ensuring the integrity and authenticity of the messages exchanged between the devices and the network server, while AppSKey is used for end-to-end encryption of the payload at the application layer. It ensures that the application data remains confidential between the end device and the application server. In addition to the node list, these two keys are also disseminated to the associated edge computing gateway by the NS, so that the traditional security mechanism can be resumed in our proposed method.

### Performance evaluation metrics

The LoRa network performance evaluation metrics include the confirmed messaging time, packet reception ratio (PRR), received signal strength indicator (RSSI), and signal-to-noise ratio (SNR). The performance of the network server which is deployed on AliCloud^44^ is evaluated in terms of CPU and memory utilization, network bandwidth, and system load.

#### Confirmed messaging time

The confirmed messaging time refers to the time interval between an end node sending confirmed data and receiving ACK from the gateway or vice versa. Numerous factors contribute to the confirmed messaging time for our edge computing gateway:

- Time required for parsing, checking, and verifying data after reception by gateway.
- Time consumed by other processes or threads on the gateway.
- Time taken by the gateway to generate the corresponding ACK.
- Parsing and verification of data by the node after receiving ACK.
- Packet transmission delay depends on the packet length and LoRa physical layer parameters.

#### PRR

The PRR is the ratio of the total number of acknowledged packets to the total number of transmitted packets. The PRR quantifies the rate of successful packet reception. It is computed as follows:

1 $$\begin{aligned} PRR = \dfrac{N_{\text{RX}}}{N_{\text{TX}}} \end{aligned}$$

where $N_\text{TX}$ is the total number of gateway transmissions, and $N_\text{RX}$ is the total number of returned ACKs.

Numerous factors affect the PRR, including communication distance, obstructions, antenna attenuation, transmitting and receiving antenna gain, and LoRa physical layer parameters of both the nodes and gateways.

#### LoRa RSSI

Equation (2) defines the RSSI^45^, which is a widely used term in various wireless technologies^46^. In our experiments, the RSSI value is read directly from the LoRa chip’s registers.

2 $$\begin{aligned} RSSI = 10lgP_\text{rx} \end{aligned}$$

where $P_\text{rx}$ denotes the received signal power in mW.

#### SNR

By comparing the signal power to the noise power, the SNR calculates the signal quality^47^. An increased SNR indicates a stronger signal, which improves transmission efficiency and signal quality. The SNR in this paper is computed using the following formula:

3 $$\begin{aligned} \begin{aligned} SNR = 10lg\frac{P_\text{SP}}{P_\text{NP}} = 20lg(\frac{A_\text{SP}}{A_\text{NP}})^2 \end{aligned} \end{aligned}$$

where $P_\text{SP}$ and $P_\text{NP}$ are the signal and noise powers, respectively. $A_\text{SP}$ denotes the amplitude of a signal in dBm, and $A_\text{NP}$ is the amplitude of noise.

#### Network server system load

In addition to the mentioned metrics to evaluate the performance of the network server, we also adopted the system load which is defined as the number of processes running on the server, including the number of processes running and waiting to run. System load can reflect how busy the server is. Specifically, the system load is rated in a range of 0 to 4 with the larger number indicating greater server resource usage. Moreover, instead of the instant system load, we focus on the average system load over a certain period, and we chose 3 periods: 1 min, 5 min, and 15 min. Then, the averaged system load denoted by $L_{t,\, p}$ for period *p* at the time instant *t* can be computed as follows:

4 $$\begin{aligned} L_{t,\, p} = \frac{1}{p}\int _{t}^{t+p}rdt \end{aligned}$$

where *r* represents the instant rate of the system load.

## Results

This section describes the obtained results of resource usage reduction on the network server and the gateway performance. In our study, we ensured comparability between tested environments by standardizing the experimental setup across all scenarios. This included using a consistent number of nodes, identical hardware specifications for the server, and a uniform software environment across tests. We simulated the network server’s load using a controlled set of applications to mimic real-world traffic and load patterns. By maintaining these consistent parameters, we aimed to accurately measure the impact of our edge-computing LoRa gateway on reducing CPU, memory, and bandwidth utilization, ensuring a fair comparison between the conventional cloud-based processing model and our proposed edge-computing approach.

### Network server resources usage reduction

When conducting experiments to evaluate how our new LoRa gateway design could alleviate the network server’s resource usage, we first used only 100 nodes. However, the results of the experiments fluctuated due to environmental factors, making it challenging to obtain the precise values of the network server’s CPU, memory, and bandwidth utilization. Conversely, using numerous nodes, such as 10,000, for the experiment can significantly increase the costs. To address these issues, the reduced resource usage of the network server was measured using simulated nodes and gateways.

**Figure 13:**
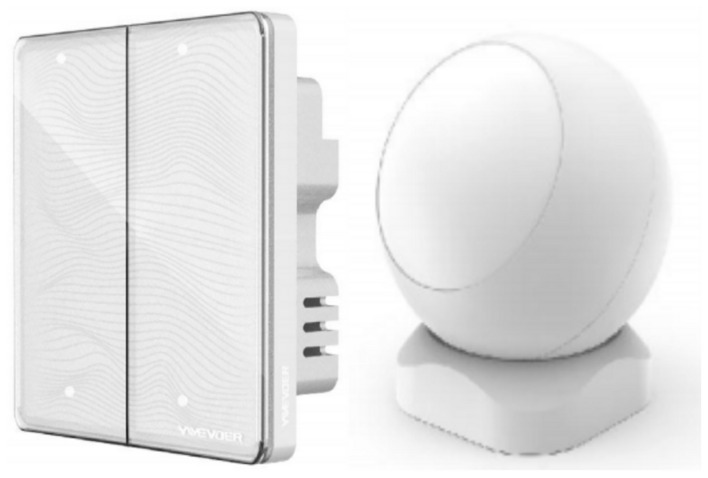
The switch and infrared sensor.

**Figure 14:**
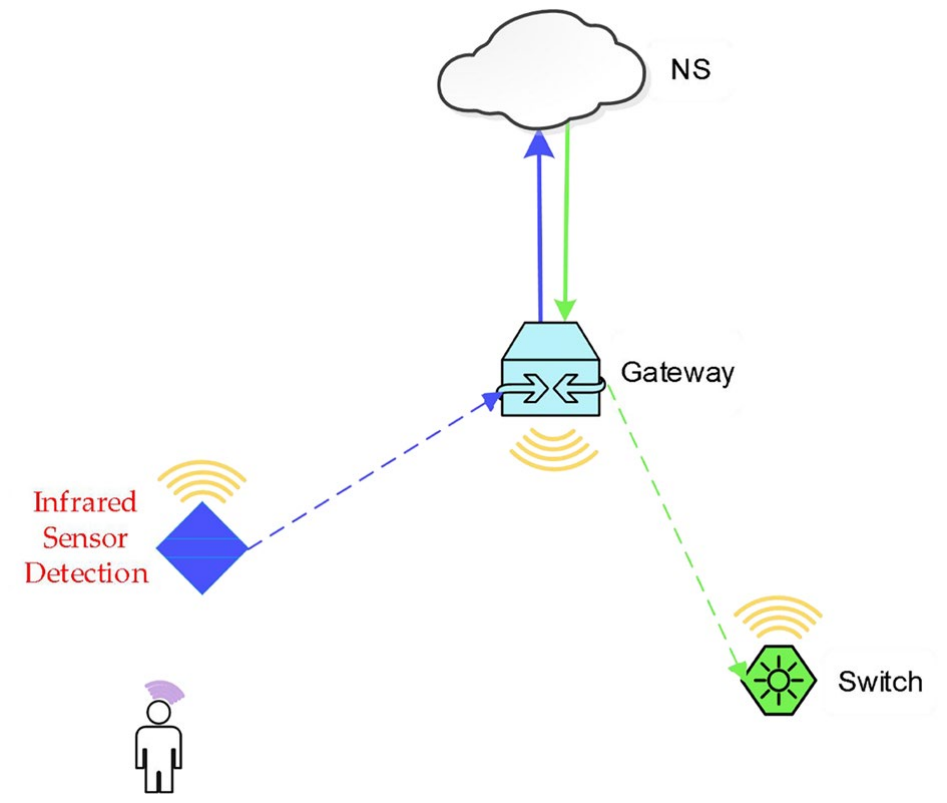
An application involving an infrared sensor, a switch, a gateway, and the network server.

Table 4 lists the configurations of the network server deployed in AliCloud. Then, we chose two categories of end nodes: switch and infrared sensor, which are shown in Fig. 13. An infrared sensor, a switch, a gateway, and the network server form an application as shown in Fig. 14. When the infrared sensor detects the movement of someone, it will transmit the movement-detected message to the gateway, which is forwarded by the gateway to the network server. After the message is received by the network server, it will send an activation command to the switch through the gateway as a response to the movement detection.

**Table 4:** NS performance configuration.

CPU	Memory (GiB)	Bandwidth (Mbps)
8-core (vCPU)	32	15

During the simulation, the numbers of switches, gateways, and infrared sensors were set to 10,000 each. As a result, they formed 10,000 concurrent applications which were divided equally into 600 concurrent groups and executed concurrently and asynchronously using multi-thread in the network server. The environment to run the simulation are shown in Table 5.

**Table 5:** Simulation environment.

CPU	System clock	Memory (RAM)	System Type
Intel(R) Core(TM) i5-7500	3.40 GHz	16.0 G	CentOS 7 x86_64

To compare the specific resource utilization of the network server, we conducted two sets of experiments. In the first set, the network server is responsible for generating both light activation commands (i.e., the application commands) and the acknowledgments for the confirmed messages sent by the infrared sensors. As a result, the gateway operates in the conventional way (i.e., transparent mode used in the current LoRaWAN standard). As a comparison, in the second set, the acknowledgments are generated by our edge computing gateway, and the network server is only responsible for the application command generation. Each experiment was conducted 100 times.

**Figure 15:**
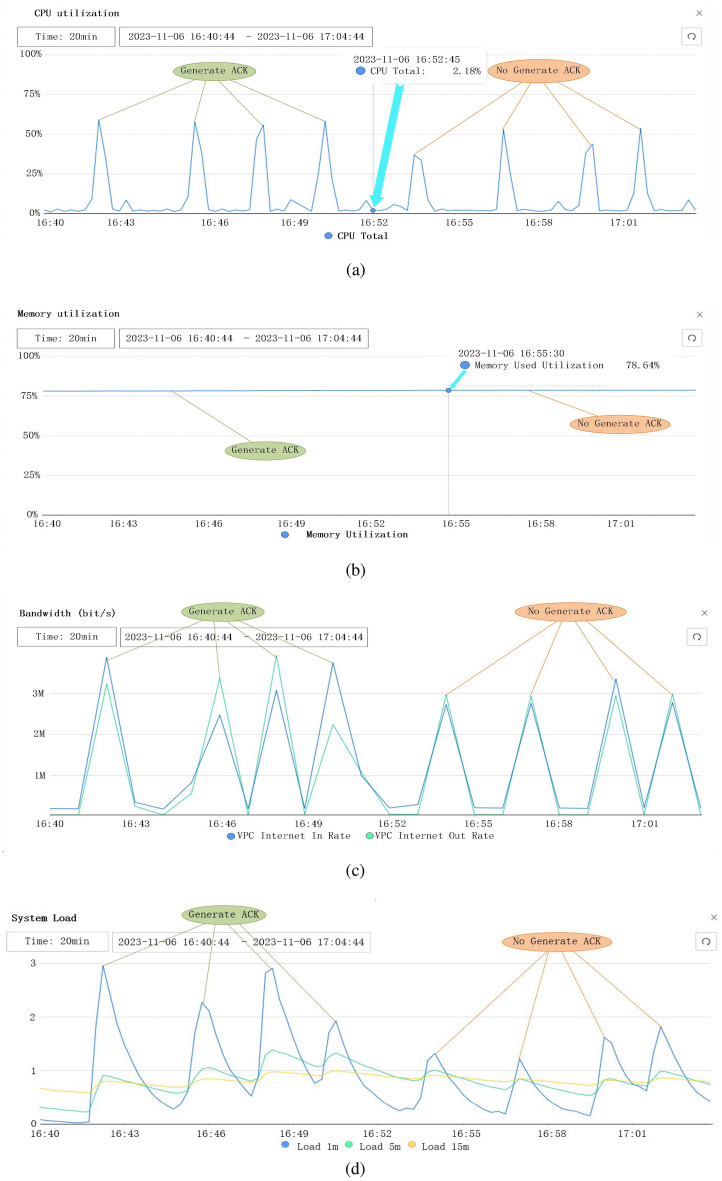
Server resource consumption. (**a**) NS CPU utilization. (**b**) NS memory utilization. (**c**) NS bandwidth utilization. (**d**) NS system load.

Figure 15a illustrates the CPU utilization of the two experiment sets. When the network server generates and sends ACKs for the 10,000 applications, the CPU utilization consistently exceeds 60%. In contrast, when the network server does not need to generate ACKs, the CPU utilization typically declines to approximately 50% or 40%. Figure 15b demonstrates that the memory utilization constantly stays at 78% with negligible fluctuations regardless of whether the network server generates and sends ACKs or not, This is due to that the network server currently has a memory of 64GB which is far more than the 10,000 applications need.

Figure 15c illustrates the bandwidth utilization for the input and output traffic when the network server whether nor not generates and sends ACKs. If it does, the peak input and output bandwidth consumption mostly exceeds 3M and can reach a level of approximately 4M. If it doesn’t, these two metrics are mostly below 3M. Figure 15d shows the averaged system load of the two experiment sets with the 3 chosen averaging periods (i.e., denoted by 1-min, 5-min, and 15-min). When the network server generates and sends ACKs, its 1-min system load can be beyond level 3, while if it doesn’t, the 1-min system load is always below level 2. If we observe the 5-min system load, generating the ACKs makes the system load of the network server slightly higher than that not. Furthermore, if the averaging period is extended to 15 min, the two experiment sets exhibit nearly identical system load, since the server is idle for the duration of 15 min.

**Figure 16:**
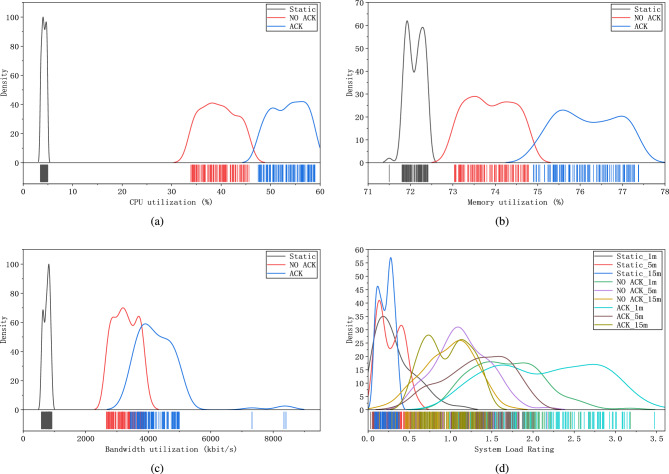
Server performance consumption for 100 Tests. (**a**) CPU utilization for 100 tests. (**b**) Memory utilization for 100 tests. (**c**) Bandwidth utilization for 100 tests. (**d**) System load for 100 tests.

In addition to the instant resource usage, we also analyzed its distribution over the entire experiment time. Figure 16a illustrates the CPU utilization distribution. As it can be seen, the CPU utilization concentrates at approximately 4.23$\%$ when no application needs to be executed (i.e., static as shown in the Fig. 16a). When the application threads without the generation of ACK are executed, the CPU utilization approximately evenly distributes in a range of 30% to 50%, with an average value of 39.46%. Conversely, when the network server generates ACKs, the CPU utilization varied between 45% and 60%, with an average of 53.51%. The difference between these two average values is 14.05%.

Figure 16b shows the memory utilization difference although it is very small. The memory usage of the static network server concentrates at approximately 72.11%. When the network server does not generate ACK, its memory utilization drops to a range of 73% to 75%, with an average of 73.88%. In the other case, memory usage increases to the range of 75% to 78%, with an average of 76.17%, which yields a difference of 2.29%, compared to the case of no ACK generated.

The bandwidth of the static network server is highly concentrated at 754.80 kbps as shown in Fig. 16c. The bandwidth usage without ACK generation varies between 2000 and 4000 kbps, with an average of 3271.27 kbps. Conversely, when the network server generates ACKs, the bandwidth usage increases to a range of 3000 to 8000 kbps, with an average of 4422.68 kbps, which yields a difference of 1151.41 kbps with the former average value.

In Fig. 16d, the set of 1-min curves reveals the remarkable system load difference between the cases of static, with, and without ACK generation. In the case without ACK generation, the system load concentrates in a level range of 1 to 2, with an average value of 1.69. However, when the ACK is generated, the system load mostly stays in the level range of 2 to 3, with an average value of 2.15. This results in a difference of 0.46 between the two values. As a comparison, the set of the 5-min curves shows that the system load difference between with and without ACK generation is much smaller than that of the 1-min. This difference continues to be smaller when the averaging period extends to the setting of 15-min. In a word, these comparisons demonstrate that employing our edge computing gateway to fulfill the acknowledgment function for the network server can greatly reduce various resource usage.

Note that the observed percentage improvements shown in Figs. 15 and 16 are the result of implementing local acknowledgment generation for end nodes, which includes parsing uplink packets, handling retransmissions, and conducting security mechanisms. Parsing acknowledgment packets directly at the gateway reduces latency and processing overhead on the network server. Locally handling retransmission minimizes the need for data packets to travel back to the network server for retransmission decisions, thereby reducing bandwidth usage and improving throughput. Integrating security mechanisms at the edge (i.e., the gateway) attains data integrity and confidentiality without imposing significant additional load on the network server.

It is also worth pointing out that based on extensive observations over a prolonged period, the inherent variability in system resource consumption (i.e., CPU, memory, and bandwidth) has been found to be very low compared to the significant improvement in these resource utilization brought by our approach. By demonstrating reductions well beyond the normal fluctuations of server resource use, our findings underscore the effectiveness of our proposed solution in achieving substantial, consistent gains in resource management, setting a new benchmark for operational efficiency in IoT network servers.

### Gateway performance

**Figure 17:**
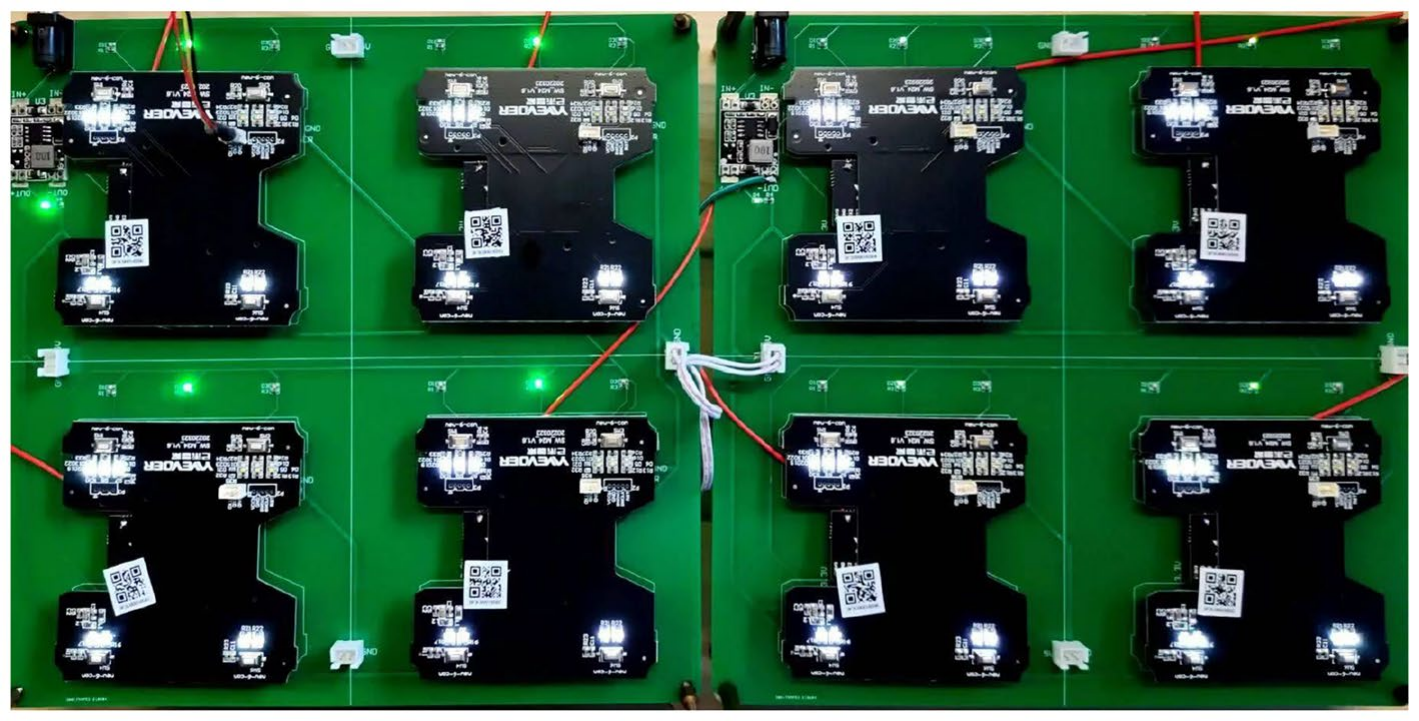
8 end nodes and the gateway.

For the gateway performance measurement, we used 8 nodes to transmit 100 packets each to a gateway, which are shown in Fig. 17.

**Figure 18:**
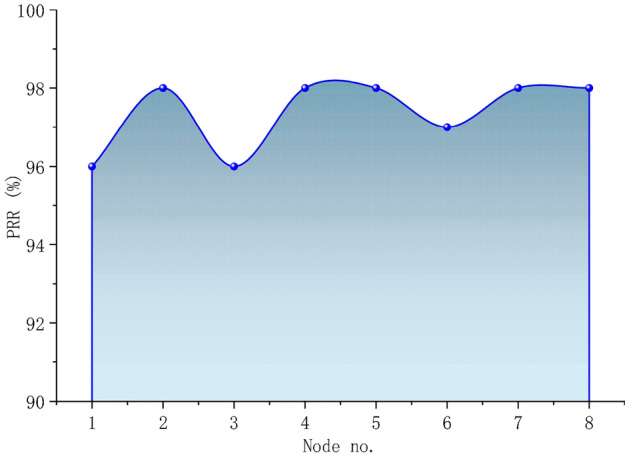
PRR.

The overall PRR results are shown in Fig. 18. Its minimum and maximum values are 96%, and 98%, respectively, and the average value is 97.38%. The PRR of a specific end node is quite similar because we placed all 8 end nodes at the same distance to the gateway.

**Figure 19:**
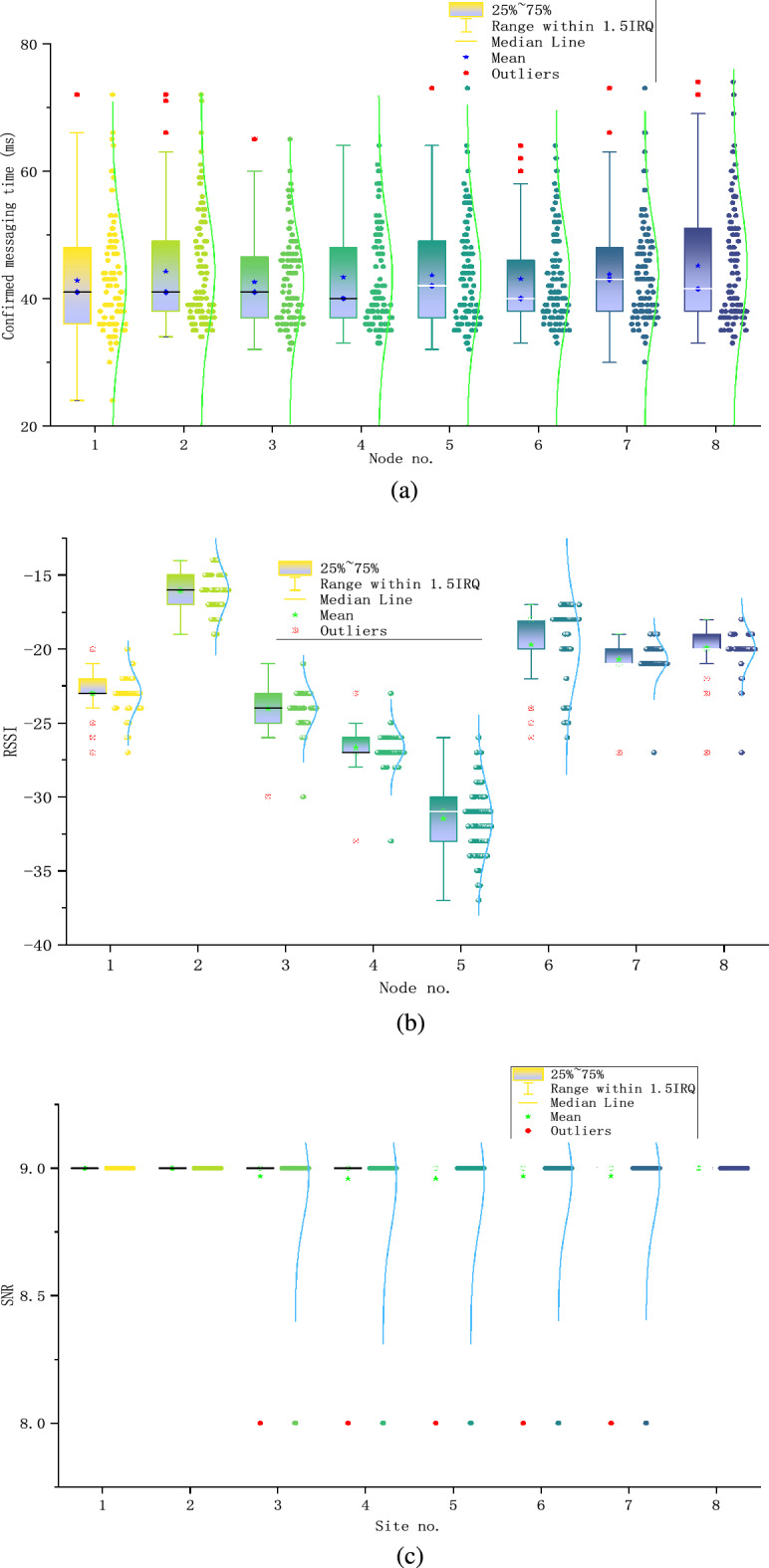
Gateway performance parameter testing. (**a**) is the PRR, (**b**) is the RTT, (**c**) is the RSSI, (**d**) is the SNR.

Figure 19a shows the confirmed messaging time, which is primarily within the range of 35 to 50 ms with an average value of approximately 43 ms. The highest distribution density of the time is around 37 ms for all end nodes. The explanation for such a phenomenon is that based on the LoRa transmission time calculation formula^27^, with the selected LoRa physical layer parameters, the node takes approximately 19 ms to send 39 bytes of data, whereas the gateway responds with a 19-bytes ACK in approximately 10 ms (i.e., 29 ms for LoRa packet time on air in total). The remaining time (i.e., approximately 8 ms) is consumed by other aforementioned tasks. In addition, it is worth noting that most of the confirmed messaging succeeded on the first attempt. Very few times are beyond 70 ms which succeeded within the second attempt. The third transmission attempt is never used during our experiment as none of the measured times is beyond 80 ms. As a comparison, in the traditional LoRaWAN Class A standard, it takes more than 6 s to complete a confirmed messaging for a maximum of 2 retransmission attempts since in each attempt, the retransmission timer expires in 2 s.

The RSSI values are illustrated in Fig. 19b, which are distributed between − 35 and − 15 dB. Despite being located in the same experimental locations, the eight nodes exhibited varying RSSI values obtained at the gateway, which can be attributed to hardware differences, such as the antennas. In contrast, the SNR value remained relatively stable at approximately 9 dB for all end nodes, which is illustrated in Fig. 19c. Such a high SNR value indicates that the signal quality between the end nodes and the gateway is very high, which is due to the close proximity between the end nodes and the gateway. The gained results are summarized in Tables 6 and 7.

**Table 6:** Resource utilization results of the traditional and proposed network servers.

	CPU peak (%)	Memory peak (%)	Bandwidth peak (kbps)	System load
Traditional LoRaWAN server	53.51	73.88	4422.68	2.15
Proposed LoRaWAN server	39.46	72.11	3271.27	1.69

**Table 7:** Communication performance results of the proposed edge-computing gateway.

PRR	Confirmed messaging time (ms)	Mean RSSI (db)	Mean SNR (db)	Cost
97.38%	43	− 22.36	9	$9.5

## Discussion

### Scalability

The proposed edge computing-enhanced LoRa gateway is primarily designed and tested within the context of a star network topology, which inherently benefits from the centralized nature of communication between end nodes and the gateway. This design facilitates direct and efficient management of data traffic, simplifies network management, and enhances scalability by allowing straightforward addition of end nodes without significantly affecting the existing network infrastructure or performance. Transitioning to a mesh network topology introduces the capability for end nodes to dynamically route messages through multiple hops to reach the gateway. While this can enhance coverage and redundancy, it also complicates network management and could impact the gateway’s performance due to increased computational overhead for handling routing information and potentially higher data traffic, which restricts the scalability of a mesh network. Note that LoRa has long-range communication capability. Thus, the multi-hop feature is not urgent, and we don’t recommend using this topology. Last, our LoRa star topology suffers the same scalability problem as bus topology, which is caused by sharing a single channel among the nodes with the gateway (i.e., packet collisions). However, this issue can be mitigated by the utilization of multiple parallel channels allocated for LoRa communications. This might be an additional task for the gateway to manage the use of the channels to avoid packet collisions.

### Comparison with Wi-Fi technology

First, the proposed LoRa gateway is specifically designed for low-power operations, making it highly suitable for applications where devices need to operate on battery power for extended periods. This contrasts with Wi-Fi routers, which are designed for high throughput and are typically powered by a continuous power supply. Second, LoRa technology provides long-range communication capabilities, allowing the proposed gateway to connect devices over distances of several kilometers in rural or open areas. This is a significant advantage over Wi-Fi, which is limited to shorter ranges, typically within tens of meters. Third, due to the low bandwidth and data rate requirements of typical LoRa applications, the proposed LoRa gateway offers a cost-effective solution for deploying IoT networks. This is particularly beneficial for applications that do not require the high data throughput provided by Wi-Fi routers.

### Hardware compatibility

The proposed edge-computing LoRa gateway is designed with a modular architecture, allowing for easy updates and integration with new technologies. This design philosophy ensures that as 5G small cell technology evolves, the gateway can be updated or adapted with minimal hardware modifications. The use of standard communication interfaces (e.g., Ethernet, SPI) and protocols (MQTT for server communication) further enhances this compatibility, as these are widely adopted in 5G infrastructure.

## Conclusion

In this paper, we have proposed and implemented an edge-computing single-channel LoRaWAN gateway using MTK7688 and SX1278 modules. In numerous emergency and control applications, real-time confirmed messaging is desired, which, however, is not supported by current gateways. To address this issue, we have proposed a novel edge computing gateway that is able to acknowledge the uplink confirmed message directly to achieve the lowest confirmed messaging time. In addition, transferring the acknowledgment function to edge-computing also relieves the CPU, memory usage, and system load on the network server. In our subsequent work, we will further integrate features such as user application functionalities and local command generation into edge-computing to enhance the edge computing capabilities of the gateway and further reduce resource usage for the network server.

## Data Availability

The datasets used and/or analysed during the current study available from the corresponding author on reasonable request.
